# Clinical significance of retinal emboli during diagnostic and therapeutic cardiac catheterization in patients with coronary artery disease

**DOI:** 10.1186/1471-2261-11-5

**Published:** 2011-01-21

**Authors:** Javad Kojuri, Morteza Mehdizadeh, Hamed Rostami, Danial Shahidian

**Affiliations:** 1 Department of Cardiology, Shiraz University of Medical Sciences,Shiraz, Iran; 2 Department of Ophthalmology, Shiraz University of Medical Sciences, Shiraz, Iran

## Abstract

**Background:**

Cardiac catheterization may cause retinal embolization, a risk factor for cerebrovascular emboli and stroke. We describe the incidence of clinically silent and apparent retinal emboli following diagnostic and interventional coronary catheterization and associated risk factors.

**Methods:**

Three hundred selected patients attending a tertiary referral center for diagnostic and therapeutic cardiac catheterization were studied. Retinal examination and examination of the visual field and acuity were done before and after catheterization by a retinal specialist.

**Results:**

There were 5 case of retinal embolus before catheterization, and 19 patients (incidence 6.3%) developed new retinal arteriolar emboli after catheterization. Only 1 patient developed clinically apparent changes in vision. Two conventional risk factors (age and hypertension) were significantly associated with new retinal emboli. The risk of retinal emboli was also significantly associated with operator expertise.

**Conclusions:**

Retinal embolism was found after coronary catheterization in 6.3% of our patients. This finding indicates that the retinal, and possibly the cerebral circulation, may be compromised more frequently than is clinically apparent as a complication of coronary catheterization. Age and hypertension are independent predictors of retinal embolism.

**Trial Registration:**

NCT01157338

## Background

Coronary atherosclerosis is the leading cause of death and disability in the developed world. Coronary angiography and angioplasty are currently the standard procedures for the diagnosis and treatment of coronary artery disease. However, these procedures are invasive and involve a risk of complications such as vascular and hemodynamic complications, contrast-related reactions, arrhythmia and myocardial infarction [1]. Moreover, because of the many opportunities for air, clots, or solid particles to enter the systemic circulation, embolism is one of the most serious complications associated with coronary catheterization [2]. The origins of systemic emboli are varied and include atheromatous emboli, cholesterol emboli (from ulcerated plaques), thrombus formation at the catheter tip [3,4], air embolism [5,6] and rarely, foreign materials from the catheter or guide wire [7].

Cholesterol emboli during catheterization are thought to be caused by the disruption of a vascular plaque (mainly by catheter manipulation) with the release of subendothelial cholesterol crystals into the blood stream [8]. One of the most dangerous consequences of cholesterol emboli is central nervous system (CNS) emboli, which can result in stroke, a rare but serious complication of cardiac catheterization [1,9]. The risk of clinically apparent stroke attributable to cardiac catheterization is reported to be between 0.11% and 0.38% [3]; however, a high incidence of post-catheterization asymptomatic cerebrovascular embolism (15%) was documented in a study that compared pre- and post- coronary catheterization brain magnetic resonance images [10]. Another study showed a 10-fold increase in the incidence of stroke among men followed for a mean of 3.4 years after an index retinal embolus over the stroke incidence seen in a control group over the same period [11]. It was also shown that persons with retinal emboli at baseline had an increased risk of dying of stroke compared to those without retinal emboli. After adjustment for systemic factors, a fatal stroke was about 3 times as likely in persons with retinal emboli during an 8-year period as in persons without retinal emboli [12].

The ophthalmic artery is the first major branch of the internal carotid artery and thus emboli from the heart and great vessels proximal to the carotid tend to directly go to the eye. Retinal arteriolar emboli are generally described as discrete plaque-like lesions in the lumen of the retinal arterioles or at bifurcations. They are composed of fibrin-platelet aggregates, cholesterol fragments, or particles of calcified valves. They originate predominately from an ulcerated atheromatous carotid artery or ascending aorta plaque, calcified aortic valves, or a mural internal carotid thrombus, and are classified as cholesterol (reflective or refractile), fibrin-platelet (dull or nonreflective) or calcific (chalky white) [13]. Retinal emboli may be a simply detectable predictor of CNS embolism. Previous studies have reported rates of retinal emboli between 55% and 100% after bypass surgery, and 1.25% and 13.2% after carotid stenting [14-16]. The rate of retinal emboli after cardiac catheterization is still controversial. Peri-coronary catheterization retinal emboli occur during or immediately after the procedure, when the femoral artery sheath is still in place [17]. Retinal cholesterol emboli, known as Hollenhorst plaques, have a distinctive focal bright, refractive yellow-orange appearance [18]. Retinal emboli are partially obstructive and asymptomatic most of the time [9]; however, central retinal occlusion, although rare, is an emergency situation whose treatment remains highly uncertain and controversial [2]. Some treatments such as intravenous mannitol or beta-blockers and oral acetazolamide do not appear to be fully effective. An experimental study of central retinal artery occlusion showed that occlusion lasting about 97 min resulted in massive, irreversible damage. Thus, no treatment instituted much longer than 1.5 hours after the onset can be expected to restore vision [19-21].

We aimed to assess the incidence of acute retinal embolism after coronary catheterization. In addition, we sought possible risk factors that may increase the possibility of this complication.

## Methods

### Study population

From February 2009 to March 2010, 300 patients who underwent diagnostic (n = 150) or therapeutic coronary catheterization (n = 150) were included in our study. There were 156 men and 144 women, and mean age was 59.36 ± 7.86 years. Exclusion criteria were arrhythmia (especially atrial fibrillation), significant valvular heart disease confirmed by transthoracic echocardiography, and peripheral vascular disease. Patients with minor coronary artery disease (stenosis less than 50%) were excluded from the study. Written informed consent was obtained from all patients, and the study was approved by the research ethic committee of the Shiraz University of Medical Sciences and Kowsar Hospital.

### Study protocol

Complete medical history and findings on physical examination were documented for each patient, and cardiovascular risk factors such as dyslipidemia, diabetes mellitus, smoking, hypertension, and obesity were recorded. Procedural data (i.e., use of balloon, size of stent, fluoroscopy time and the total duration of the procedure excluding fluoroscopy time) were recorded. The number of occluded vessels and degree of stenosis were recorded.

Coronary catheterization via a femoral access point was performed by 5 experienced cardiologists, each with at least 4 years of experience in cardiac catheterization. In all patients who underwent diagnostic angiography, standard coronary angiography was done according to the Seldinger method, with a 6F catheter and guide wire. No heparin was given for diagnostic angiography. For percutaneous transluminal coronary angioplasty with stent implantation with or without balloon dilation, a 6F or 7F catheter was used. These patients were given 10 000 international units of heparin as a bolus to maintain an activated clotting time of more than 250 s, and received at least 360 mg aspirin and 600 mg clopidogrel bisulfate before the angioplasty. A standard nonionic contrast medium (Omnipaque or Visipaque, GE, Buckinghamshire, UK or Ultrvist, Bayer HealthCare Pharmaceuticals, Leverkusen, Germany) was used. For diagnostic angiography, sheaths were removed immediately after the procedure. In patients who underwent therapeutic catheterization, the sheaths were removed 6 h after the procedure.

### Eye examination

One day before the procedure, visual acuity was determined with the Snellen chart and visual field was measured with the confrontation test and subjective history. The visual field and visual acuity were checked again 24 h after the procedure. Retinas were examined by a retinal subspecialist. Pre-catheterization (median 19 h, range 13-25 h) and post-catheterization examinations (45 h, 17-96 h) were done by a retinal specialist after pupil dilation with 1.0% tropicamide. The macula and posterior pole were examined with a 78D non-contact lens through a slit lamp, and indirect ophthalmoscopy with a Volk 2.2D lens was done to visualize the peripheral portions of retina. We did not use digital retinal photography because this technique cannot visualize the retina beyond 60 degrees, so small peripheral emboli would be missed.

### Statistical analysis

For statistical analyses, the patients were classified into two groups: those with and those without retinal emboli. For categorical and noncategorical variables, we used the chi-squared test to determine whether differences between groups were significant. A *P *value < 0.05 was considered significant.

## Results

### Final study population

Of the 408 patients initially recruited, cardiac catheterization was performed in 406, but only 300 patients attended their post-catheterization eye examination. Twenty patients had normal angiograms or mild coronary artery disease, and the others refused further cooperation. Therefore, this report is based on statistical analysis of the data for 300 patients (mean age 59.36 ± 7.86 years, 156 men and 144 women).

### Risk factors

Our analysis is based on just 300 patients, so the present study lacks sufficient power to draw firm conclusions about differences in risk factors between the two groups. Most risk factors such as dyslipidemia, male sex, smoking, diabetes mellitus and body mass index did not differ significantly between patients with and without new retinal emboli (Table 1). Stent length, stent diameter, fluoroscopy time, total duration of the procedure, and balloon insertion before the intervention had no effect on the incidence of new emboli (Table 2). Severity of coronary artery disease had no effect on the rate of retinal emboli, but age and hypertension were significantly associated with the appearance of new retinal lesions (Table 1). We also found a significant correlation between operator expertise and the risk of new retinal emboli (Table 2).

**Table 1:** Characteristics of 300 patients in Shiraz, Iran, with and without new retinal emboli after cardiac intervention.

Characteristics	Post-procedure results		
	Normal (n = 281)	New lesions (n = 19)	*P*
Age	59 ± 7.83	64.5 ± 6.16	0.018
Sex			
Male	146	10	0.955
Female	135	9	0.955
Smoking	43	2	0.573
Diabetes mellitus	87	7	0.593
Hypertension	146	15	0.022
Hyperlipidemia	135	10	0.698
Body mass index >30	43	3	0.955

**Table 2:** Procedural risk factors, severity of coronary artery disease, and their relation to new retinal emboli in 300 patients in Shiraz, Iran.

Characteristics	Post-procedure results		
	Normal (n = 281)	New lesion (n = 19)	*P*
Stent length	32.23 ± 13.8	32 ± 17.6	0.591
Stent width	2.87 ± 0.34	2.65 ± 0.2	0.504
Balloon insertion before process	73	6	0.897
Duration of fluoroscopy	8.69 ± 3.94	8.26 ± 2.8	0.871
Duration of procedure	16.11 ± 4.7	19.21 ± 4.28	0.188

### Eye examination

Retinal examination by the retinal specialist showed that 5 patients (1.7%) had prior retinal emboli: 3 in the angiography (diagnostic) group (2%) and 2 in the angioplasty (therapeutic) group (1.33%). All of them were cholesterol emboli. These numbers were too small for valid statistical comparison. Post-procedure retinal examination diagnosed a total of 19 new lesions (6.33%). Of these 19 patients, 8 had undergone cardiac catheterization for diagnostic purposes (5.33%) and the remaining 11 (7.33%) for therapy.

**Figure 1:**
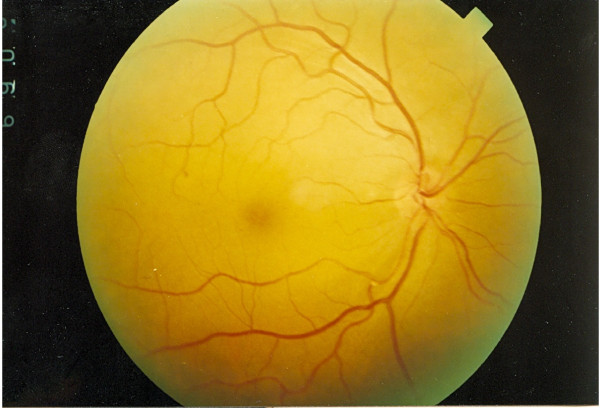
**A new retinal embolus in the main branch of the retinal artery after catheterization **.

There was no significant difference between groups in the location of emboli in the right or left eye (10 lesions in the right eye and 9 in the left; *P *> 0.05). Seven of 19 lesions were located in the upper temporal field, 5 in the lower temporal, 5 in the lower nasal and the remaining 2 in the upper nasal field. All lesions were small and peripheral and caused only partial obstruction except in one case, a 72-year-old woman who complained of a large scotoma in the visual field of her right eye. On examination she had normal visual field and acuity, but ophthalmoscopic investigation revealed central emboli with partial obstruction in the main branch of the retinal artery (Figure 1).

Visual field and acuity were tested within 24 h after catheterization. The findings of all post-procedure visual assessments were negative.

### Catheterization procedures

Our search for likely procedural risk factors failed to identify any potential relationship between risk factors and the appearance of retinal emboli. Although we observed, in this small series, a slightly higher incidence of retinal emboli in patients who underwent catheterization for therapy rather than diagnosis only, this difference was not significant (*P *= 0.477).

### Fluoroscopy time and duration of the procedure

We found no significant difference in fluoroscopy time between patients with and without new retinal emboli (P = 0.871).

## Discussion

Of the clinical and procedural variables analyzed in our sample of 300 patients who underwent diagnostic or therapeutic catheterization, 2 conventional risk factors (age and hypertension) and operator expertise were strongly associated with the appearance of new retinal emboli. Other conventional and procedural risk factors showed no significant relation with the appearance of this lesion. At the start of the study, we hypothesized a strong association between the severity of coronary artery disease and the risk of retinal embolization. We reasoned that more severe coronary artery disease would be related with more atherosclerosis of the aorta, which in turn would make patients more susceptible to retinal emboli. As noted by Segal and colleagues, plaque broken off from the ascending aorta or the aortic arch can be the main source of systemic emboli including retinal lesions [3]. It appears likely that the risk of vascular plaque mobilization increases with aging and hypertension.

Klein et al. [12] found that the prevalence of baseline retinal emboli was associated with higher pulse pressure, hypertension, cardiovascular disease, diabetes mellitus and past and current smoking. The prevalence of baseline retinal emboli in their patients was 1.3%. Our study identified some of the same risk factors, including age and hypertension, as playing a role in post-procedure retinal emboli. A prospective cohort study by Thyer et al. [22] found a prevalence of baseline retinal emboli of 5%, but obtained no evidence suggesting that coronary catheterization contributes to retinal embolism shortly after the procedure. Funduscopic examination detected no new retinal emboli in 97 patients who underwent coronary catheterization. The differences between their findings and ours may have arisen, in part, because Thyer and colleagues included patients with a normal angiogram in their sample of patients who underwent coronary catheterization, and their sample was too small to reveal significant differences.

Kreis et al. [23] found a 2% incidence of acute retinal embolism after coronary catheterization, and indicated that the retinal and possibly cerebral circulation may be more severely compromised more frequently than is clinically apparent. In their study the main method of retinal examination was digital retinal photography. New retinal emboli after cardiac catheterization were seen in 6.3% of our patients. The reason for our higher figure compared to the incidence found by Kreis and colleagues may be that routine retinal photography is not sensitive enough to detect tiny retinal emboli that cause partial, peripheral arteriolar obstruction.

Busing et al. [10] found that diagnostic and interventional cardiac catheterization increased the risk of silent cerebral infarction to 15%. Their sample of 48 patients was not large enough to analyze risk factors, and they noted that the statistical analysis of their results was weak, so they could not evaluate potential risk factors in detail.

Our figures for the incidence of retinal emboli after coronary catheterization are based on the findings of retinal examination by a retinal specialist. Nineteen patients developed retinal emboli, only one of which was clinically apparent. With regard to the rate of emboli formation and their clinical significance, cardiac catheterization can be considered a safe procedure. In our patients, all lesions were located in non-eloquent areas of the retina (one was found in the main branch of the central retinal artery) and were too small to be clinically apparent. However, when lesions affect more important areas of the retina, such as the central retinal artery, they can have severe clinical consequences. Cardiologists who perform coronary catheterization should be cognizant of the clinical symptoms and signs of large retinal emboli (i.e., any visual complaint, especially a decrease in visual field or acuity), because they can have a devastating effect on the retina. Because coronary catheterization may contribute to retinal artery occlusion, prompt consultation with an ophthalmologist should be considered if symptoms suggestive of visual disturbance appear during or soon after coronary catheterization.

The rate of retinal embolization appeared to be slightly higher after therapeutic cardiac catheterization than after diagnostic angiography; however, this difference was not statistically significant. We suspect that further manipulation of the aortic root might increase the risk of embolism, and further research will be needed to test this hypothesis. Retinal emboli occurring after cardiac catheterization warrant a careful vascular workup including a search for the emboli in the aortic arch, the great vessels and the chambers of the heart itself. Vascular ultrasound, transesophageal echocardiography and (potentially) computed tomographic angiography or magnetic resonance angiography can be extremely useful in this regard. These techniques, unfortunately, were not available at our center when we studied this cohort.

## Conclusion

Our findings in a selected cohort of patients suggest that cardiac catheterization is safe and feasible. Retinal emboli are generally clinically silent and do not produce any visual sequelae; however, age and hypertension are independent predictors of retinal emboli.

## Competing interests

The authors declare that they have no competing interests.

## Authors' contributions

JK is corresponding author, main researcher, catheterization facilitator and one of angiography doers. MM is ophthalmologist and examed all retinas. HR and DS are data collectors and contacted the patients for their follow up and filled the forms and performed data analysis. HR provided the preliminary format of article under supervision of JK. All authors read and approved the final manuscript.

## Pre-publication history

The pre-publication history for this paper can be accessed here:

http://www.biomedcentral.com/1471-2261/11/5/prepub
